# The role of training in student examiner rating performance in a student-led mock OSCE

**DOI:** 10.1007/s40037-020-00643-8

**Published:** 2020-12-22

**Authors:** Jian Hui Koo, Kim Yao Ong, Yun Ting Yap, Kum Ying Tham

**Affiliations:** 1 Singapore General Hospital, Singapore, Singapore; 2 Tan Tock Seng Hospital, Singapore, Singapore; 3 Lee Kong Chian School of Medicine, Nanyang Technological University, Singapore, Singapore

**Keywords:** OSCE, Peer assisted learning

## Abstract

**Introduction:**

Peer assessments are increasingly prevalent in medical education, including student-led mock Objective Structured Clinical Examinations (OSCE). While there is some evidence to suggest that examiner training may improve OSCE assessments, few students undergo training before becoming examiners. We sought to evaluate an examiner training programme in the setting of a student-led mock OSCE.

**Methods:**

A year‑2 mock OSCE comprised of history taking (Hx) and physical examination (PE) stations was conducted involving 35 year‑3 (Y3) student examiners and 21 year‑5 (Y5) student examiners who acted as reference examiners. Twelve Y3 student-examiners attended an OSCE examiner training programme conducted by senior faculty. During the OSCE, Y3 and Y5 student examiners were randomly paired to grade the same candidates and scores were compared. Scores for checklist rating (CR) and global rating (GR) domains were assigned for both Hx and PE stations.

**Results:**

There was moderate to excellent correlation between Y3 and Y5 student examiners for both Hx (ICC 0.71–0.96) and PE stations (ICC 0.71–0.88) across all domains. For both Hx and PE stations, GR domain had poorer correlation than CR domains. Examiner training resulted in better correlations for PE but not Hx stations. Effect sizes were lower than the minimum detectible effect (MDE) sizes for all comparisons made.

**Discussion:**

Y3 student examiners are effective substitutes for Y5 student examiners in a Y2 mock OSCE. Our findings suggest that examiner training may further improve marking behaviour especially for PE stations. Further studies with larger sample sizes are required to further evaluate the effects of dedicated examiner training.

## Introduction

Peer assessment in medical education has become more prevalent and popular in recent years. Traditionally, this has been used to gather more quantitative information on trainee’s performance [1]. The benefits of peer assessment are both to the student being assessed, who is given more opportunities to practice, and to the assessor, who is able to learn through teaching in a process known as assessment for learning (AfL) [2]. In addition, having a larger pool of examiners for formative assessments may alleviate faculty teaching burden in an environment where there is an increasing medical student population and limited teaching resources. As peer assessment becomes more commonplace, an emerging area of interest involves the role of student examiners in Objective Structured Clinical Examinations (OSCEs).

A 2014 systematic review revealed that one of the more well-studied modalities of peer assessment is in student-led OSCEs [3]. Mock OSCEs are simulations of clinical examinations involving physical examination or history taking with standardised patients and senior students as examiners. Mock OSCEs have been found to be reasonable alternatives in providing additional practice for medical students while also allowing them more opportunities for early intervention [4–7]. Various topics such as history taking, physical examination and patient communication are commonly tested. During these mock OSCEs, examiners typically assign checklist ratings (CR) and global ratings (GR) to participants [8–10]. The CR assess one aspect of a participant’s behaviour in the OSCE while GR describe the overall impression of a participant’s performance.

Previous studies comparing year‑4 (Y4) or year‑5 (Y5) medical students and faculty examiners showed that senior medical students are comparable to doctors as OSCE examiners [11, 12]. Particularly for GR, peer examiners typically have moderate to high agreement and positive correlations with faculty examiners [13]. In terms of CR, the reliability of peer examiners is less clear. Peer examiners may award lower, higher or similar CR depending on the station [13]. For instance, some studies suggest that junior examiners with less experience as examiners (e.g. medical students or junior doctors) are more lenient [14, 15]. Others suggest that they are stricter [16], and some found no significant difference [17]. Despite efforts to standardise features of the OSCE to make it more objective, examiner marking behaviour remains a significant source of variation in scores [18, 19].

It has been suggested that better OSCE assessment is likely if medical students undergo training before taking on the role of examiners [14], and that examiner training should be directed at both teaching skills and OSCE-specific assessment skills [20]. In addition, evaluation should be carried out to determine the type and amount of training necessary for each specific OSCE [21]. Van der Vleuten recommended that in order for peer assessment to be effective for learning, it should be approached as a complex professional skill that requires intensive training [1]. In a study on faculty examiners, those who had undergone training performed more consistently in their rating of students in an OSCE when compared to untrained faculty examiners [22]. Despite this evidence, there exists a critical gap in existing literature, with few peer examiners in previous studies having undergone training prior to assessment.

With this gap in knowledge, it would be of interest to compare untrained and trained peer examiners in their performance as peer assessors in a mock OSCE. In this study, we examine whether junior year‑3 (Y3) student examiners are as effective as senior year‑5 (Y5) student examiners in performing as peer examiners and whether undergoing training will improve the marking behaviour of the Y3 student examiners compared to their seniors. We hypothesise that:

1. Y3 student examiners will be as effective as Y5 student examiners in a year‑2 (Y2) mock OSCE, and
2. Y3 student examiners who have undergone examiner training will grade more similarly to Y5 student examiners in their assessment compared with untrained examiners.

## Methods

### Participants

This study was conducted at the Lee Kong Chian School of Medicine, Nanyang Technological University in Singapore.

Three groups of examiners were recruited:

1. Y3 student examiners who underwent examiner training (*n* = 12),
2. Y3 student examiners who did not undergo examiner training (*n* = 23), and
3. Y5 student examiners (*n* = 21).

All Y3 student examiners were in their first year of clinical rotations with no prior experience as mock OSCE examiners. The Y3 student examiners were randomised into two groups. Group 1 (*n* = 12) attended an OSCE examiner training session a month prior to the mock OSCE and an hour-long examiner calibration session on OSCE day. Group 2 (*n* = 23) only attended the examiner calibration session. All Y5 student examiners attended the examiner calibration session. OSCE examiner training was the active intervention under study with Group 2 acting as the control arm.

A prior scoping study showed that when assessing student’s performance, senior peers should be used preferentially over more junior peers [4]. In this setting, the Y5 student examiners (*n* = 21) acted as reference examiners, with the assumption that final year students have the knowledge and skills to be effective examiners for a Y2 mock OSCE. All Y5 student examiners had already passed their final Bachelor of Medicine and Bachelor of Surgery examinations. They had experience as examiners in previous mock OSCEs, with informal training in the form of examiner calibration before each OCSE.

### OSCE examiner training

A half-day examiner training session was conducted by a senior faculty member who was experienced in teaching medical students, examining OSCE and conducting OSCE examiner training for doctors.

The session comprised training on assessment of Hx and PE stations with emphasis on (a) the expected behaviour that competent Y2 candidates should demonstrate, (b) how to calibrate examiners to agree with what constitutes a pass for specific domains in the marksheet and (c) how to use the marksheet. Adapted from prior studies, three domains were identified for Hx stations (two CR domains—content and communication, and a GR domain) and two domains were identified for PE stations (one CR domain—execution, and a GR domain) [15].

The Y3 student examiners then completed a practicum by assessing Y2 candidates in standardised PE and Hx stations. This was followed by feedback discussions involving both Y5 and Y3 student examiners giving feedback to Y2 candidates. Although not within the scope of this study, feedback discussions have been shown to be beneficial to the learning of both candidates and peer examiners [12].

### Structured marksheet

A global score for both PE and Hx stations was graded on a 5-point Likert scale (poor, borderline, adequate, good, excellent) and was intended to capture the examiner’s overall impression of the student’s performance. For Hx stations, the content domain was graded based on the candidate’s ability to elicit relevant points in the history, while the communication domain assessed the candidate’s rapport and communication skills with the standardised patient. Both were marked on a 5-point scale. Similarly, the execution domain for PE stations assessed the candidate’s ability to carry out a full physical examination using the same 5‑point scale. The novel tool was adapted based on the school’s marking rubric for OSCEs which uses the same 5‑point Likert scale, and modified based on prior studies and input from senior teaching faculty [12].

### Mock OSCE design

One month after the examiner training, the mock OSCE comprising 4 Hx and 3 PE stations was conducted for Y2 candidates. Student examiners assembled earlier and completed the calibration session that emphasised the content that candidates needed to cover.

Multiple circuits of the mock OSCE were conducted, with different Y3 and Y5 student examiners and Y2 candidates during each circuit. As there were fewer Y5 than Y3 student examiners, each Y5 student examiner examined approximately eight Y2 candidates each, while each Y3 student examiner examined approximately five Y2 candidates each. The Y3 and Y5 student examiners were randomly paired and assigned to either a PE or Hx station. Student examiners used standardised domain-rating structured marksheets identical to those used in the OSCE examiner training. Both examiners were in the same room to assess each candidate concurrently but independently. Hence, each Y2 candidate would receive two scores, one from the Y3 student examiner and one from the Y5 student examiner.

### Data analysis

Mirroring the practice in our school, each domain was assigned a numerical score. For each candidate, the domain scores and global scores were calculated. Descriptive statistics were employed to compare means, standard deviations (SD) and Cohen’s *d* effect size. Normality of data was analysed with the Shapiro-Wilk test. Continuous non-normal data were analysed with the Mann-Whitney U test with significant threshold set at 5% (*p* < 0.05). Minimum effect sizes were estimated for all comparisons using G*Power 3.1 software.

Intra-class correlation coefficients (ICC) were calculated based on a 1-way mixed effects model [23]. Agreement across the following dyads was explored for both PE and Hx stations:

- Group A: Trained Y3 student examiners versus Y5 student examiners
- Group B: Untrained Y3 student examiners versus Y5 student examiners

The levels of correlation were interpreted as follows: <0.4: poor agreement; 0.4–0.75: moderate agreement; >0.75: excellent agreement [24].

All statistical analyses were performed on SPSS (Version 25) for Macintosh.

### Ethics

All candidates and student examiners participated voluntarily and gave written consent.

## Results

A total of 104 Y2 candidates took part in the mock OSCE, with 108 and 66 attempts for Hx and PE stations, respectively.

### Physical examination

Tab. 1 summarises the correlation between scores given by Y3 and Y5 student examiners and mean scores and effect sizes given for each domain in PE stations.

**Table 1:** Intraclass correlation coefficient and mean scores of Y3 and Y5 student examiners for PE stations

**Intraclass Correlation Coefficients**				
			**Execution Score**	**Global Score**
Group A: Trained Y3 vs. Y5 examiners (*n* = 26)			0.96	0.81
Group B: Untrained Y3 vs. Y5 examiners (*n* = 40)			0.85	0.71
**Mean scores**				
	**Execution (Mean** **±** **SD)** **(max** **=** **100)**	**Cohen’s** ***d***	**Global (Mean** **±** **SD)** **(**max **=** **5)**	**Cohen’s** ***d***
**Group A: Trained Y3 vs. Y5 examiners**				
Y3 examiners	55.30 ± 16.70	0.08	3.64 ± 0.82	0.23
Y5 examiners	56.60 ± 16.90		3.46 ± 0.71	
**Group B: Untrained Y3 vs. Y5 examiners**				
Y3 examiners	57.70 ± 15.60	0.13	3.53 ± 0.69	0.25
Y5 examiners	55.80 ± 12.50		3.71 ± 0.75	

Overall, there was excellent correlation between the Y3 and Y5 student examiners in the execution domain across both Groups A and B (ICC = 0.96, 0.81). Lower, but still moderate–excellent correlations between Y3 and Y5 student examiners were observed in the global domain in both Groups (ICC = 0.85, 0.71). Scores given by Y5 student examiners were more highly correlated with trained Y3 (Group A) compared to untrained Y3 student examiners for both execution (ICC = 0.96 vs 0.85) and global domains (ICC = 0.81 vs 0.71).

When looking at mean domain scores, trained Y3 student examiners awarded lower scores for execution but higher scores for global domain compared to Y5 student examiners. The opposite was true for untrained Y3 student examiners, who awarded higher scores for execution and lower scores for global domain. However, overall, effect sizes for all comparisons remained low. Minimum detectible effect (MDE) sizes for comparisons in Group A were 0.81 and MDE sizes for comparisons in Group B were 0.65.

### History taking

Tab. 2 summarises the correlation between Y3 and Y5 student examiners and mean scores given for each of the domains in Hx stations.

**Table 2:** Intraclass correlation coefficient and mean scores of Y3 and Y5 student examiners for Hx stations

**Intraclass Correlation Coefficients**						
	**Content Score**		**Communication Score**		**Global Score**	
Group A: Trained Y3 vs. Y5 examiners (*n* = 53)	0.81		0.74		0.71	
Group B: Untrained Y3 vs. Y5 examiners (*n* = 55)	0.88		0.77		0.72	
**Mean scores**						
	**Content (Mean** **±** **SD)** **(max** **=** **100)**	**Cohen’s** ***d***	**Communication (Mean** **±** **SD)** **(max** **=** **100)**	**Cohen’s** ***d***	**Global (Mean** **±** **SD)** **(max** **=** **5)**	**Cohen’s** ***d***
**Group A: Trained Y3 vs. Y5 examiners**						
Y3 examiners	47.20 ± 14.30	0.36	66.20 ± 21.90	0.51	3.68 ± 0.61	0.44
Y5 examiners	52.40 ± 14.20		55.90 ± 18.70		3.39 ± 0.70	
**Group B: Untrained Y3 vs. Y5 examiners**						
Y3 examiners	42.30 ± 16.80	0	59.50 ± 23.60	0.10	3.46 ± 0.71	0.03
Y5 examiners	42.30 ± 14.70		57.30 ± 20.70		3.44 ± 0.85	

Overall, there was moderate to excellent correlation between Y3 and Y5 student examiners for content (ICC = 0.81, 0.88), communication scores (ICC = 0.74, 0.77), and global scores (0.71, 0.72). Unlike the PE stations, untrained Y3s had higher correlation with Y5 student examiners in all domains tested.

The mean content scores for trained Y3 student examiners were lower than for Y5 student examiners, while untrained Y3 and Y5 student examiners had the same mean scores. For communication and global scores, both trained and untrained Y3 student examiners gave higher scores than Y5 student examiners. Effect sizes for the comparisons in Group A were larger than those in Group B, but all were lower than the MDE sizes. MDE sizes for comparisons in Group A were 0.56 and MDE sizes for those in Group B were 0.55.

## Discussion

Previous studies have established the role of medical students as OSCE examiners [12]. This study was designed to compare the performance of junior peer examiners with more senior peer examiners and to see if training would further improve their performance. Our findings showed that overall correlations between Y3 and Y5 student examiners were moderate to excellent in both the PE and Hx stations. When broken down into domains, correlation between Y3 and Y5 student examiners was generally higher in CR domains compared to GR domains across both PE and Hx stations. The effect of training on correlation between Y3 and Y5 student examiners was mixed, with improvement in correlation only in PE stations but not in Hx stations. Comparison of mean scores showed generally small effect sizes between examiners, and all were smaller than the minimum detectible effect sizes that this study was powered to detect.

The high correlations seen between the Y3 and Y5 student examiners support our hypothesis that Y3 student examiners are as effective as their seniors when grading a mock OSCE for more junior Y2 students. This builds upon previous studies which have shown that senior medical student examiners are equivalent to faculty examiners in OSCEs that test basic medical skills [11]. Of note, correlations seen in this study were higher than those of previous studies [13]. One explanation is that most previous studies looked at student-faculty correlations, and it is possible that the use of junior and senior peer examiners in this study resulted in higher student-student correlations.

When comparing the subtypes of OSCE stations, PE stations had higher correlations compared to Hx stations and this has been observed in other studies as well. Physical examination stations may be less susceptible to measurement error [15] because the steps are prescriptive and techniques are well documented [25]. There is therefore little room for examiners’ subjective interpretation when assessing a candidate’s PE performance. Essentially candidates who perform all the necessary steps in the PE station would receive good scores even if examiners were less experienced. This is especially so in a mock OSCE where candidates examine standardised patients who do not have abnormal findings and are not assessed on their detection and interpretation of findings.

The overall correlations for the Hx stations are lower than PE stations. This is in keeping with the findings from a previous study whereby history taking and clinical communications are more susceptible to examiners’ interpretation [15]. Effective communication for information gathering is closely related to personal style and may be affected by examiners’ preferred style. Nonetheless, the Y3s appeared to be effective examiners as evidenced by moderate–excellent correlations across all domains.

For both Hx and PE stations, the correlation between Y3 and Y5 student examiners was the poorest for GR regardless of examiner training. Previous studies also found relatively poor agreement between novice examiners and trained examiners on global pass/fail decisions [26]. It is suggested that novice examiners may have more difficulty in assigning accurate global scores due to lack of experience and therefore have poorer judgment of a candidate’s overall performance [27, 28]. In addition, the mean global scores given by Y3s were higher than corresponding Y5s in all groups except for untrained Y3s grading the PE stations. This is a finding seen in previous studies as well, where peer examiners typically grade higher in GR domains [13, 29].

The effect of training on the effectiveness of Y3 student examiners was less clear.

For PE stations, trained Y3s had higher correlations with Y5s compared with the untrained Y3s. However, correlations between trained Y3s and Y5s in the Hx stations were lower across all domains. In addition, the effect sizes of differences in mean scores in both groups A and B were smaller than the minimum detectible effect. This presented a limitation in the analysis of comparison between Y3 and Y5 student examiners. Due to the limited sample sizes and low effect sizes, the authors were unable to definitively conclude that training resulted in Y3 student examiners grading more similarly to Y5 student examiners.

Nonetheless, current evidence seems to suggest that training positively impacts examiner behaviour in various domains. Improved correlations between trained Y3 and Y5 student examiners in the PE stations seem to suggest that training junior students makes them more effective examiners. This could possibly be due to the more prescriptive nature of examining PE stations, which requires looking out for certain steps that are performed and relies less on clinical judgement.

Effects of training on scores in the Hx stations were more mixed. Lower correlation in the content domains could be due to lack of focus of the training programme on content of the individual Hx stations, and more on general examiner skills. Future studies could consider implementing training which is more specific to the content of individual exam stations.

Although our study did not evaluate the effect of examiner training on assessing non-verbal communication, previous authors found that non-verbal expression may be the domain most impacted by training [30]. This might be particularly relevant to OSCE stations such as counselling on breaking bad news, where both verbal and non-verbal communication play key roles.

### Future direction

In a previous scoping review on peer examination in OSCEs, most studies reviewed had some form of examiner training, but this was largely limited to training on use of a standardised marking sheet [13]. We believe that future studies assessing training peer examiners should include dedicated training sessions such as the one described in this study. More studies should also be done to compare the effectiveness of these dedicated training sessions to fill this gap in existing literature.

## Conclusion

This study shows that in an OSCE testing basic medical skills, junior Y3 student examiners are effective substitutes for more senior Y5 student examiners. Scores in CR domains were comparable between Y3s and Y5s, while Y3s typically gave higher GR scores than Y5s. The effect of training remains unclear from this study, but likely results in juniors marking more similarly to senior student examiners especially in physical examination stations. Future work should include dedicated training sessions for peer examiners in addition to same-day examiner calibration in order to better evaluate the impact of training peer examiners.
